# Blastocyst Complementation: The New Kid on the Xenotransplantation Block

**DOI:** 10.1111/xen.70165

**Published:** 2026-09-06

**Authors:** Mark B. Nottle, Peter J. Cowan, Wayne J. Hawthorne

**Affiliations:** ^1^ Robinson Research Institute and School of Pharmacy and Biomedical Science Adelaide University Adelaide South Australia Australia; ^2^ Immunology Research Centre St. Vincent's Hospital Melbourne Victoria Australia; ^3^ Department of Medicine University of Melbourne Melbourne Victoria Australia; ^4^ Translational Transplantation Therapies Program Westmead Institute for Medical Research Westmead New South Wales Australia; ^5^ Department of Surgery, School of Medical Sciences University of Sydney Westmead Hospital Westmead New South Wales Australia

**Keywords:** blastocyst complementation, embryo, gene editing, pluripotent stem cells, xenotransplantation

## Abstract

The transplantation of genetically modified pig organs and tissues is arguably the most promising approach to overcoming the world‐wide shortage of organ and tissues for transplantation. The advent of gene editing has seen a resurgence in interest in this approach which has also allowed blastocyst complementation to be explored as an alternative to solving this challenge. Genetic strategies are increasingly being used to explore this possibility and overcome the many scientific hurdles this presents. While blastocyst complementation faces many scientific challenges the ethical questions surrounding this approach are equally important and will need to be addressed at the same time, if this new kid on the xenotransplantation block is to become a reality.

## 1 Introduction

The transplantation of genetically modified pig organs and tissue is arguably the most promising solution to overcoming the world‐wide shortage of organ and tissues for transplantation [1]. The advent of gene editing has led a resurgence in interest over the past decade in this approach which has seen the first pig to human kidney and heart transplants as well as the approval of clinical trials [1]. Gene editing is also allowing blastocyst complementation, the production of pigs with human organs and tissues to be explored, providing a new approach to solving this challenge [2].This involves knocking out key developmental genes for example PDX‐1 the key transcription factor required for pancreatic development, using gene editing in the developing embryo and injecting human pluripotent stem cells to fill this void to produce the missing pancreas [2]. Proof of concept has already been demonstrated in mouse and rat models [3, 4], and has been extended to the pig where the contribution of human pluripotent stem cells to various developmental lineages has been demonstrated [5].

## 2 Scientific Hurdles

The major scientific hurdle blastocyst complementation faces currently, appears to be the lack of incorporation and differentiation of human pluripotent stem cells following their injection into the developing embryo [2]. The reasons for this are likely to be many and varied, and genetic strategies are increasingly being developed to improve this [2]. This includes manipulating interspecies cell adhesion [6] as well as expressing anti‐apoptotic and pro‐growth factors [7]. Interestingly, we showed over two decades ago that successful incorporation of pig inner cell mass cells (used as a model for embryonic stem cells) into blastocysts depends largely on the genetic sex of the injected cells with germline transmission being 100% when XY cells are injected into XX and XY embryos compared with 20% and 0% when XX cells are injected into these embryos respectively [8]. While freshly isolated inner cell mass cells are not pluripotent stem cells, sexing of these would seem reasonable in developing blastocyst complementation.

## 3 The Need for Pig Allotransplantation Models

These and other questions are possibly best addressed initially in a pig allotransplantation model such as the Westran pig, which has been bred for xenotransplantation research [9]. This has been inbred over numerous generations to the extent that allo‐transplanted skin grafts are not immediately rejected. The other advantage of this and other breeds such as the Auckland Pig, compared with for example mini pigs, is their larger in size making their organs compatible with that of humans [9]. Successful complementation using porcine pluripotent stem cells also remains a critical milestone for advancing the field as successful complementation of an empty developmental niche has only been convincingly demonstrated using porcine blastomeres [10]. Such a model could be used to readily address the question of what type of pluripotent cell is best in terms of incorporation [2, 5]. This is important because the human pluripotent stem cell lines (including induced pluripotent stem cell lines), used initially for blastocyst complementation studies resemble primed mouse embryonic stem cells [2, 5]. These are derived post implantation and do not readily make chimaeras in mice, suggesting they are mismatched in terms of their development with the developing embryo [2, 5]. In contrast, naïve embryonic stem cells which are isolated from the preimplantation epiblast and by their very definition produce chimaeras, are likely to improve incorporation as demonstrated in more recent studies [5, 11]. This could be readily examined in an allotransplantation pig model as a range of pluripotent stem cells including extended pluripotency and naïve lines, have now been derived for the pig [12].

## 4 The Spectre of Rejection

The lack of incorporation of injected cells is obviously related to the evolutionary distance between pigs and humans and the many physiological challenges that this presents [2]. This lack of incorporation may also mean that cells are being rejected even at this early stage because of immunological incompatibility. This would explain why incorporation of human pluripotent stem cells has only been demonstrated early in gestation [5], which is also around the time the immune system is being established in the pig [12]. This includes when the first leukocytes, macrophages and initial lymphoid cells are detected in the fetal liver [13]. Such a xenogenic barrier has recently been proposed with phagocytic macrophages playing an initial role [14]. The “reverse engineering” of genetic strategies aimed at overcoming immune rejection in xenotransplantation may therefore be relevant in developing blastocyst complementation. Alternatively, cells may have achieved tolerance by the time the immune system is established. Either way it would seem that this question needs to be addressed sooner than later for blastocyst complementation to advance further.

## 5 Legal and Ethical Challenges

While numerous scientific hurdles remain to be overcome, the legal and ethical challenges blastocyst complementation present are equally important. What xenotransplantation has taught us is that these need to be addressed at the same time as the scientific challenges, if this approach is to be accepted. In many if not most countries, legal restrictions are already in place regarding the production of human and animal chimaeras following the birth of Dolly the sheep and the possibility of human cloning [15]. However, these restrictions vary between countries and even states. The restrictions this place on research in many countries, are significant [15]. In other countries such as Japan, legislation has already been revised to allow blastocyst complementation to be explored [15]. Similarly, the moral and ethical questions that blastocyst complementation present will be challenging, in particular the possibility of neurological and germline chimaeras [14]. Importantly, genetic strategies to overcome such eventualities are already being developed [16, 17].

## 6 Conclusion

While much research remains to be done for blastocyst complementation to become a reality, it is worth noting xenotransplantation suffered similar scepticism in the 1980's [1]. However, in what is a relatively short time in research terms, we have now witnessed the first kidney and heart transplant in humans and the approval of clinical trials in the USA [1]. While these achievements are allowing blastocyst complementation to be explored, the public acceptance xenotransplantation now enjoys will need to replicated if blastocyst complementation is to become a reality.

## Conflicts of Interest

MBN is Director/owner of ICMStemcell Pty Ltd.
